# Cell-type-specific neuroanatomy of cliques of autism-related genes in the mouse brain

**DOI:** 10.3389/fncom.2015.00055

**Published:** 2015-05-29

**Authors:** Pascal Grange, Idan Menashe, Michael Hawrylycz

**Affiliations:** ^1^Department of Mathematical Sciences, Xi'an Jiaotong-Liverpool UniversitySuzhou, China; ^2^Department of Public Health, Faculty of Health Sciences, Ben-Gurion University of the NegevBeer-Sheva, Israel; ^3^Allen Institute for Brain ScienceSeattle, WA, USA

**Keywords:** computational neuroanatomy, gene expression, cerebellum, cell types, autism spectrum disorder

## Abstract

Two cliques of genes identified computationally for their high co-expression in the mouse brain according to the Allen Brain Atlas, and for their enrichment in genes related to autism spectrum disorder (ASD), have recently been shown to be highly co-expressed in the cerebellar cortex, compared to what could be expected by chance. Moreover, the expression of these cliques of genes is not homogeneous across the cerebellar cortex, and it has been noted that their expression pattern seems to highlight the granular layer. However, this observation was only made by eye, and recent advances in computational neuroanatomy allow to rank cell types in the mouse brain (characterized by their transcriptome profiles) according to the similarity between their spatial density profiles and the spatial expression profiles of the cliques. We establish by Monte Carlo simulation that with probability at least 99%, the expression profiles of the two cliques are more similar to the density profile of granule cells than 99% of the expression of cliques containing the same number of genes (Purkinje cells also score above 99% in one of the cliques). Thresholding the expression profiles shows that the signal is more intense in the granular layer. Finally, we work out pairs of cell types whose combined expression profiles are more similar to the expression profiles of the cliques than any single cell type. These pairs predominantly consist of one cortical pyramidal cell and one cerebellar cell (which can be either a granule cell or a Purkinje cell).

## 1. Introduction

The neuroanatomical structures underlying autism spectrum disorder (ASD) traits are the subject of intense research efforts, as ASD is one of the most prevalent and highly heritable neurodevelopmental disorders in humans (Newschaffer et al., 2007; Amaral et al., 2008; Levy, 2009; Lord, 2011). Recent genomic advances have led to the association of more than 300 candidate genes with ASD susceptibility (Jacquemont et al., 2006; Szatmari et al., 2007; Cook and Scherer, 2008; Basu et al., 2009; Gilman et al., 2011; Kumar et al., 2011; Levy et al., 2011; Myers et al., 2011; Anney et al., 2012; Iossifov et al., 2012; Neale et al., 2012; O'Roak et al., 2012; Sanders et al., 2012). However, the list is far from closed and the molecular mechanisms and brain regions underlying ASD traits remain largely unclear. While no major anatomical pathology has been observed in brains of ASD cases, various molecular and neuroimaging studies have linked several brain regions to ASD. The cortex is often associated to capacities, such as planning and interpreting language, that are impaired in autism. Indeed, differences in gene expression patterns in the cortex of ASD brain have been found in Voineagu et al. (2011). On the other hand, the cerebellum, which appeared much earlier in evolution than the cortex, is a less likely candidate for implication in autism, because it is more often associated to motor skills. However, a strong body of evidence, in particular from the from the study of post-mortem autistic brains (Skefos et al., 2014), suggests an implication of the cerebellum in ASD. Moreover, the cerebellum may be connected to remote parts of the brain, including the neocortex involved in cognitive development and social interaction (see Wang et al., 2014).

The mouse model has recently benefited from an unprecedented flow of data, which begs for computational analysis. Important sources of data include:

1. genetic databases of ASD, such as AutDB (Basu et al., 2009; Kumar et al., 2011);
2. gene-based maps: the Allen Brain Atlas (ABA) of the adult mouse (Ng et al., 2005, 2007a,b, 2009; Lein et al., 2006; Sunkin and Hohmann, 2007; Lee et al., 2008; Hawrylycz et al., 2011a,b), which consists of thousands of brain-wide *in situ* hybridization (ISH) gene-expression profiles, digitized, and co-registered to the Allen Reference Atlas (ARA) (Dong, 2008);
3. cell-based maps: the ongoing development of a classification of cell types in the mouse brain based on their transcriptome profiles (Arlotta et al., 2005; Chung et al., 2005; Sugino et al., 2005; Rossner et al., 2006; Cahoy et al., 2008; Doyle et al., 2008; Heiman et al., 2008; Okaty et al., 2009, 2011).

These sources of data are complementary to each other. Recently, we used the ABA to examine the spatial co-expression characteristics of genes associated with ASD susceptibility in the AutDB database (Menashe et al., 2013). We identified two networks of highly co-expressed genes that are enriched with autism genes and significantly overexpressed in the cerebellar cortex. These results added to the mounting evidence of the involvement of the cerebellum in autism (Vargas et al., 2005; Lotta et al., 2014). However, the complex internal structure of the cerebellum requires a further investigation of the specific cerebellar regions or cell types associated with ASD.

On the other hand, cell-type-specific transcriptomes were recently combined with the ABA in order to estimate the brain-wide density of cell types (Grange et al., 2014), using a linear mathematical model, which amounts to decomposing the gene expression data of the ABA over a set of measured cell-type-specific transcriptomes (see also Ko et al., 2013; Tan et al., 2013 for cell-type-specific analyses of the ABA, and Abbas et al., 2009 for a similar mathematical approach in the context of blood cells). These estimates have potential application to the neuroanatomy of ASD: whenever a brain region exhibits over-expression of ASD-related genes, this region can also be compared to the neuroanatomical patterns of cell types, revealing which cell types are involved. Computational neuroanatomy has so far combined the AutDB and the ABA one one hand (Menashe et al., 2013), and cell-type-specific transcriptomes and the ABA on the other hand (Grange et al., 2014). In this paper we will close this loop by looking for computational links between ASD-related genes from AutDB and cell-type-specific transcriptomes.

It was observed in Menashe et al. (2013) that two cliques of co-expressed autism genes appear to be overexpressed in the granular layer of the cerebellum. However, this observation was based on visual comparison of the expression patterns of the genes in these two cliques to sections of the estimated density patterns of cell types^1^. This approach by mere visual inspection is far from satisfactory since it does not make use of the computational potential of the ABA (Bohland et al., 2010; Grange and Mitra, 2012; Grange et al., 2013). Moreover, post-mortem studies of brains of autistic patients (Skefos et al., 2014) have shown alterations in the Purkinje layer of the cerebellum, rather than in the granule cells.

In the present study we re-examine the two cliques discovered in Menashe et al. (2013) using recent developments of computational neuroanatomy relating cell-type-specificity of gene expression to neuroanatomy. We extend the Monte Carlo methods developed in Menashe et al. (2013) (to estimate the probability of co-expression among a set of genes) to the comparison between the expression of a set of genes and the spatial density profile of a cell type. This allows to estimate the probability of similarity between gene-expression profiles of cliques and spatial distributions of all cell types considered in Grange et al. (2014). Finally, we look for linear combinations of pairs of density profiles of cell types that are more similar to the expression profiles of cliques of genes than any single cell type.

## 2. Methods

### 2.1. Cosine similarity between the expression profile of a clique of genes and the density of a cell type

#### 2.1.1. Cliques of genes

We re-examine the brain-wide expression profiles of the two cliques 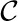
_1_ and 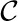
_2_ of genes identified in Menashe et al. (2013) based on their exceptional co-expression properties, which consist of 33 and 6 genes, respectively:

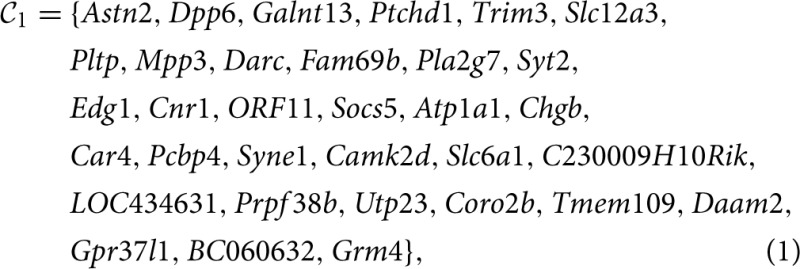

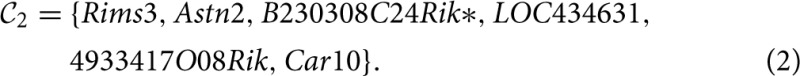





They both contain genes from the AutDB database (Basu et al., 2009; Kumar et al., 2011) of ASD-related genes(*Ptchd1, Galnt13, Dpp6* and *Astn2* for the first clique, *Astn2* and *Rims3* for the second).

#### 2.1.2. Gene expression energies from the Allen Brain Atlas

The adult mouse brain is partitioned into *V* = 49,742 cubic voxels of side 200 microns, to which ISH data are registered (Lein et al., 2006; Dong, 2008) for thousands of genes. For computational purposes, these gene-expression data can be arranged into a voxel-by-gene matrix^2^. For a cubic voxel labeled *v*, the *expression energy* of the gene *g* is a weighted sum of the grayscale-value intensities evaluated at the pixels intersecting the voxel:

(3) $$\begin{array}{l} E(v,g)=\text{expression energy of gene labeled }g\\ \text{ in voxel labeled }v, \end{array}$$

Like the analysis of Grange et al. (2013) and Menashe et al. (2013), the present analysis is restricted to the coronal ABA, for which the entire mouse brain was processed in the ABA pipeline (whereas only the left hemisphere was processed for the sagittal atlas).

#### 2.1.3. Cell-type-specific microarray data and estimated cell-type-specific density profiles

The cell-type-specific microarray reads collated in Okaty et al. (2011) from the studies (Arlotta et al., 2005; Chung et al., 2005; Sugino et al., 2005; Rossner et al., 2006; Cahoy et al., 2008; Doyle et al., 2008; Heiman et al., 2008; Okaty et al., 2009) (for *T* = 64 different cell-type-specific samples) are arranged in a type-by-gene matrix denoted by *C*, such that

(4) $$\begin{array}{l} C(t,g)=\text{expression of gene labeled }g\\ \text{ in cell type labeled }t, \end{array}$$

and the columns are arranged in the same order as in the matrix *E* of expression energies defined in Equation (3). In Grange et al. (2014), we proposed a simple linear model for a voxel-based gene-expression atlas in terms of the transcriptome profiles of cell types and their spatial densities:

(5) $$E(v,g)=\sum_{t}\rho_{t}(v)C(t,g)+\text{Residual}(v,g),$$

where the index *t* denotes the *t*-th cell type, and ρ_*t*_(*v*) denotes its density at voxel labeled *v*. The profile ρ_*t*_ is a spatial density, to be distinguished from the expression profile of a fixed cell type across all genes. More precisely, the values of the cell-type-specific density profiles were computed in Grange et al. (2014) by minimizing the value of the residual term in Equation (5) over all the (positive) density profiles, which amounts to solving a quadratic optimization problem (with positivity constraint) at each voxel:

(6) $${\left(\rho_{t}(v)\right)}_{1\leq t\leq T}={\text{argmin}}_{\nu \in R_{+}^{T}}\left(\text{​}\sum_{g}\text{​}\left(E(v,g)-\sum_{t=1}^{T}\nu (t)C(t,g)\text{​}\right){\text{​}}^{2}\text{​}\right)\text{ ​}.$$

The solution of this problem at every voxel happens to be quite sparse (with fewer than 6 distinct cell types detected at most voxels). Adding a term proportional to the *L*^1^-norm of ν in the above objective function can increase sparsity (adapting the search for marker genes implemented in Grange et al. 2013), but the diversity of cell types present in a given voxel is expected to be larger in reality than in our model, and should be increased if the model is refitted to a richer panel of cell-type-specific transcriptomes. However, if data sets increase to dramatically higher values than *T* = 64, *L*^1^-penalization could become necessary to increase sparsity (or to match it with known results in well-studied voxels).

#### 2.1.4. Cosine similarity between spatial gene-expression patterns and cell-type-specific spatial density patterns

The quantitative study of spatial co-expression of genes in Menashe et al. (2013) combines the columns of the matrix of gene-expression energies (Equation 3) by computing the cosine similarities of all pairs of genes in the cliques 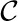
_1_ and 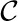
_2_. These cosine similarities are then compared to those obtained from random sets of genes containing the same numbers of elements as 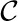
_1_ and 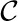
_2_, respectively. This technique can be adapted to compare brain-wide gene-expression profiles to the spatial density of cell types, simply by considering cosine similarities between gene-expression profiles and cell-type-specific density profiles.

Given a set 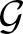 of genes from the coronal ABA (selected either computationally based on their co-expression properties, or based on curation of the biomedical literature, for instance 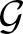 = 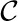
_1_ or 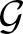 = 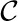
_2_), we can compute the sum of their expression profiles:

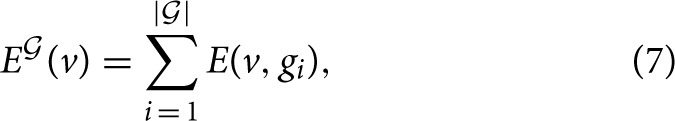



where *g*_*i*_ is the column index in the matrix of expression energies (Equation 3) corresponding to the *i*-th gene in the set 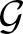, and |
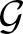
| denotes the number of genes in this set. The quantity *E*^
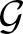
^ is an element of **R**^*V*^_+_, just as the brain-wide density profile of a cell type estimated from Equation (6). We can therefore estimate the similarity between *E*^
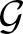
^ and the density of cell type labeled *t* by computing the cosine similarity

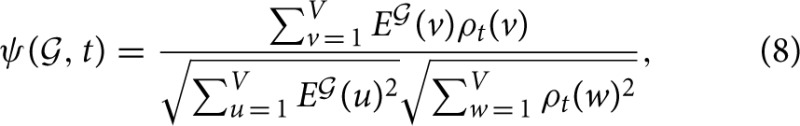



which is a number between 0 and 1 by construction.

Our model *assumes* that various sources of noise result in an additive term. However, the efficacy and stability of the biological agent binding to mRNA can vary from gene to gene, resulting in multiplicative noise. The model of Equation (5) assumes that the expression energies depend linearly on the quantity of mRNA present at each voxel (ignoring saturation effects for strong expression), and with a gene-independent coefficient (ignoring multiplicative noise). Multiplicative noise could have a strong influence when studying the sum of expression profiles of several genes in a clique. However, it was checked in Menashe et al. (2013) that the two cliques of genes in the present study are over-expressed in the cerebellar cortex, even if gene-expression profiles are separately normalized before the sum (Equation 7) is performed, which reduces the influence of multiplicative noise.

#### 2.1.5. Statistical significance of the similarity between expression profiles of genes and density profiles of cell types

Furthermore, for a fixed cell type, we can estimate how exceptional the similarity ψ(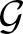, *t*) is, compared to what would be expected from random sets of |
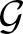
| genes drawn from the coronal ABA. This is a finite problem, but it becomes hugely complex in a regime where |
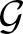
| is relatively large but still small compared to the size of the entire atlas (which is the case for both cliques in the present study). We can take a Monte Carlo approach, draw *R* random sets of |
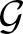
| genes and simulate the cumulative distribution function (CDF) of the cosine similarity^3^ between the expression profile of a random set of |
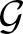
| genes and the density profile of cell-type labeled *t* (this CDF depends only on the cell type and on the number of genes |
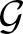
|, so we can denote it by CDF_*t*, |
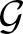
_|). By the law of large numbers, we obtain an estimate of this CDF by taking an average of *R* random sets, and the probability 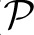
_*R*_(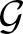, *t*) of getting a lower value of cosine similarity than ψ(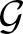, *t*) after *R* random draws converges to the true probability when *R* is large enough (in the present case the problem is finite, see Menashe et al., 2013 for details of the method).

The precision of our estimates depends on the value of *R*. We can use Hoeffding's inequality to control the probability of being within a known error from the true CDF, as a function of the number *R* of random draws. As we are estimating the probability of having larger cosine similarity than expected by chance by summing *R* Bernoulli variables, Hoeffding's inequality (see Hastie et al., 2009 for instance) states that for any τ, the probability of missing the true value of the probability 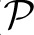
(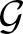, *t*) by τ is bounded in terms of τ and the number of random draws *R* as follows:

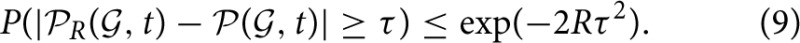



For instance, taking τ = 0.01 and *R* = 26,500 leads to a value of 0.01 for the bound on the r.h.s. of the inequality (Equation 9), so it is enough to draw this number of random sets of genes to obtain an estimator within 1 percent of the true probabilities, with probability at least 99 percent.

Having conducted the simulation of the distribution of cosine similarities for a choice of *R* based on Hoeffding's inequality, we can rank cell types for a fixed clique 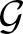 by decreasing values of statistical significance:

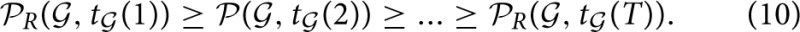



#### 2.1.6. Similarity between thresholded gene-expression energies and cell-type-specific densities

Given that the expression profiles of the cliques of interest in this study is much less sparse than any of the densities of cell types estimated in Grange et al. (2014), the genes in the cliques must be expressed in several different cell types, but there are large differences in expression between cortical voxels and cerebellar voxels for instance, and also within the cerebellar cortex (see **Figures 2A,B**). We propose to threshold brain-wide expression profile of each clique, and to recompute the cosine similarities with density profiles, in order to discover which neuroanatomical cell-type-specific patterns are highlighted with more intensity. If the profile of a given cell type is highlighted by a given clique, when the threshold grows from zero to low values of the threshold, the cosine similarity is expected to grow, since many voxels with low values of expression energy, that penalize the cosine similarity to the cell type, are put to zero by the threshold. Let us denote by τ the value of the threshold. We compute the thresholded expression energies of the cliques and cosine similarities as follows:

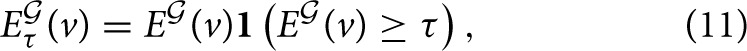

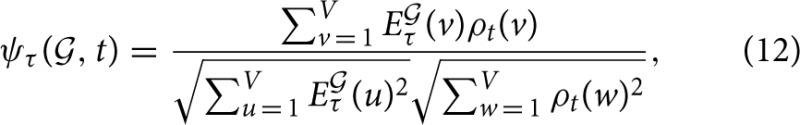





At very large values of the threshold, expression energies are going to be put to zero everywhere, and the cosine similarities decrease to zero (for all cell types). So the cosine similarity between the expression of the two gene cliques and the cell types they highlight are expected to exhibit peaks when plotted as a function of the threshold. The higher the peak, and the higher the corresponding value of the threshold, the more intensely the cell type is highlighted.

### 2.2. Cosine similarity between expression of a clique of genes and the density of a pair of cell types

Instead of ranking single cell types by the significance of the similarity between their density profile and the (possibly thresholded) expression of a given clique, we can extend our analysis to combinations of cell types. The simplest modification of our similarity analysis consists of a search for *pairs* of cell types whose combined density profile is more similar to the expression profile of a given clique than any single cell type in the data set.

As the density profiles of two cell types labeled *t*_1_ and *t*_2_ are two vectors in the voxel space **R**^*V*^, they define a plane in voxel space, and they provide a base of this plane (provided the two vectors ρ_*t*_1__ and ρ_*t*_2__ are linearly independent, which is the case for all pairs (*t*_1_, *t*_2_) in the present study). To characterize how well the expression profile of a clique of genes coincides with the reunion of two given cell types labeled *t*_1_ and *t*_2_, we have to solve the following minimization problem:

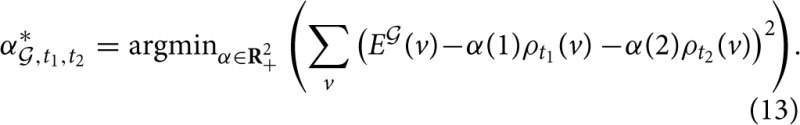



This problem is analogous to the one stated in Equation (6), but it corresponds to fitting one vector in voxel space by linear combination of two vectors with positive coefficients, not *V* vectors in gene space using *T* vectors with positive coefficients. Having solved this problem for a given pair of cell types labeled by (*t*_1_, *t*_2_), we know the closest vector to the clique 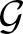 that can be obtained by combining these two cell types. We can compute the cosine similarity between this optimal vector and the expression profile of the clique (and denote it by ψ(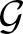, *t*_1_, *t*_2_), which symbol will be used in **Table 3** and in the caption of **Figure 5**). We can repeat this computation in order to obtain a Monte Carlo simulation of this cosine similarity, just as we did in the case of single cell types. More precisely, we compute the closest vector to *E*^
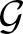
^ in the plane of voxel space spanned by ρ_*t*_1__ and ρ_*t*_2__, which we denote by 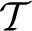
_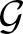, *t*_1_, *t*_2__:

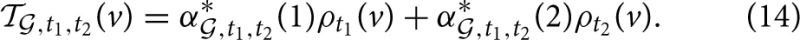



The cosine similarity between this optimal vector and the expression vector *E*^
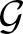
^ is readily computed as:

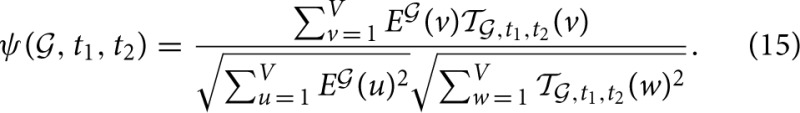



Having computed this quantity for a given clique 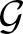 and all pairs of cell types in our data set, we can detect the pairs of cell types for which the optimization of Equation (13) leads to the largest improvement in cosine similarity, for instance by providing a better fitting than any single cell type. This motivates us to consider the following pairs of cell types:

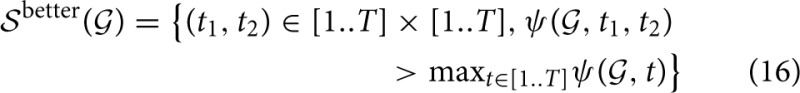



Again, for a given pair of cell types, the value of the cosine similarity can be biased by the size of the support of the two underlying cell types, but we can estimate the probability of getting a lower cosine similarity from random cliques of genes (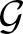
_1_, 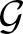
_2_, …, 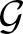
_*R*_, with |
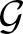
| genes each), simply by repeating the computation of cosine similarities (Equation 8), with the optimal vector 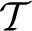
_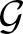, *t*_1_, *t*_2__ (defined in Equation 14) substituted to the single density profile:

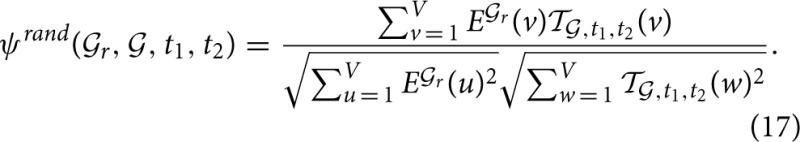

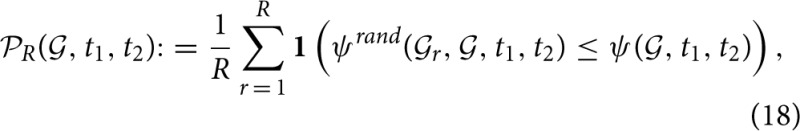





where *R* can again be worked out for given thresholds using Hoeffding's inequality.

### 2.3. Cosine similarity between a brain-wide density profile and a brain region

Given a brain region ω defined in the ARA, we define the normalized vector χ_ω_ in voxel space whose non-zero entries correspond to the voxels belonging to the region ω:

(19) $$\chi_{\omega }(v)=\frac{1(v\in \omega )}{\sqrt{\sum_{w\;=\;1}^{V}1{\left(w\in \omega \right)}^{2}}}.$$

Given a density profile ρ_*t*_, we can compute its cosine similarity to χ_ω_, in the same way that was used in Menashe et al. (2013) with gene-expression profiles:

(20) $$\phi_{\omega }(\rho_{t})=\frac{\sum_{v\;=\;1}^{V}\rho_{t}(v)\chi (v)}{\sqrt{\sum_{w\;=\;1}^{V}\rho_{t}{\left(w\right)}^{2}}}.$$

The quantities ϕ_ω_(*E*^
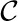
_1_^) and ϕ_ω_(*E*^
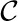
_2_^) were shown in Menashe et al. (2013) to be exceptionally large compared to quantities obtained from cliques of the same size, when ω is taken to be the cerebellar cortex. In this study the quantity ϕ_ω_ will be used to study the neuroanatomy of density profiles of cell types shown to be highly similar to expression profiles (see **Figure 7** for sorted values of ϕ_ω_(ρ_*t*_) with ω taken to be the cerebral cortex).

## 3. Results

### 3.1. Granule cells and Purkinje cells are the most significantly similar cell types to both cliques

**Figure 1:**
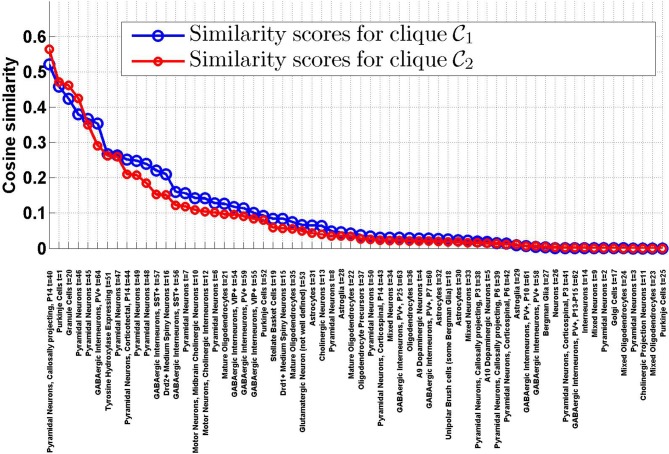
**Similarity scores between the** ***T*** **= 64 densities of cell types in our data set and the cliques 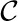
_1_ and 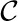
_2_, sorted by decreasing order of ψ(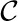
_1_,** ***t*****)**. There is a good agreement between the rankings of cell types induced by the two cliques. The first four cell types in both cliques are labeled *t* = 40 (pyramidal neurons), *t* = 1 (Purkinje cells), *t* = 20 (granule cells), *t* = 46 (pyramidal neurons).

We computed the cosine similarities between the expression profiles of the two cliques 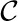
_1_ and 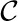
_2_ and the density profiles of the *T* = 64 cell types estimated in Grange et al. (2014), using Equation (8). The sorted values are plotted on Figure 1. It appears that the ranking of the cell types by cosine similarity is roughly conserved between the two cliques, and that no more than a third of the cell types have a cosine similarity of more than 10% to either clique.

For each cell type, we computed the probabilities 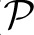
_*R*_(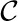
_1_, *t*) and 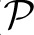
_*R*_(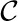
_2_, *t*) for *R* = 27,000 random sets (of 33 genes each for clique 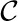
_1_, of 6 genes each for clique 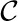
_2_). Tables 1, 2 show the cell types for which the cosine similarity is larger than 10%, ordered by decreasing values of statistical significance. For both cliques, granule cells (labeled *t* = 20) and Purkinje cells (labeled *t* = 1), have the highest value of *P*_*R*_ (more than 99% for both cliques in the case of granule cells). For each of the two cliques, one more cell type has a value of *P*_*R*_ larger than 80% (mature oligodendrocytes, labeled *t* = 21, in the case of 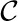
_1_, pyramidal neurons, labeled *t* = 46, in the case of 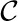
_2_). The statistical significance (i.e., the value of 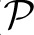
_*R*_) drops sharply after the third rank for both cliques. Our computational analysis therefore returns a list of four cell types to which at least one of the two cliques in this study is significantly similar (more similar than at least 80% of the sets of genes in our Monte CArlo simulations).

**Table 1:** **Table of cell types sorted by decreasing values of statistical significance for clique 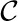
_1_ (see Equation 10), measured by the probability 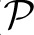
**_***R***_**(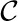
_1_,** ***t*****), for** ***R*** **= 27,000**.

**Cell type**	**Rank by significance, *t*^−1^_ 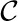 _1__(*t*)**	**Index *t***	** 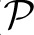 _*R*_( 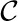 _1_, *t*), (%)**	**ψ( 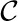 _1_, *t*), (%)**
Purkinje cells	1	1	100	45.9
Granule cells	2	20	100	42.4
Mature oligodendrocytes	3	21	99.5	12.7
GABAergic interneurons, PV+	4	64	38.4	35.3
GABAergic interneurons, PV+	5	59	37.6	11.3
GABAergic interneurons, SST+	6	57	36.1	22.1
GABAergic interneurons, SST+	7	56	34.8	16
GABAergic interneurons, VIP+	8	54	33.7	11.8
Tyrosine hydroxylase expressing	9	51	29.3	26.7
GABAergic interneurons, VIP+	10	55	26.4	10.1
Drd2+ medium spiny neurons	11	16	25.4	21
Motor neurons, cholinergic interneurons	12	12	20.9	14.2
Motor neurons, midbrain cholinergic neurons	13	10	18.5	14.2
Pyramidal neurons	14	6	9.5	12.8
Pyramidal neurons	15	7	1	15.6
Pyramidal neurons, corticotectal, P14	16	44	0.6	25.2
Pyramidal neurons	17	49	0.4	24.8
Pyramidal neurons, callosally projecting, P14	18	40	0.4	52.1
Pyramidal neurons	19	48	0.4	23.9
Pyramidal neurons	20	46	0.2	37.9
Pyramidal neurons	21	45	0	36.6
Pyramidal neurons	22	47	0	26.4

Only cell types for which the cosine similarity is larger than 10% are shown.

**Table 2:** **Table of cell types sorted by decreasing values of statistical significance for clique 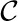
_2_ (see Equation 10), measured by the probability 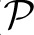
**_***R***_**(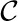
_2_,** ***t*****), for** ***R*** **= 27,000**.

**Cell type**	**Rank by significance, *t*^−1^_ 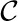 _2__(*t*)**	**Index *t***	** 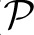 _*R*_( 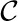 _2_, *t*), (%)**	**ψ( 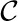 _2_, *t*), (%)**
Granule cells	1	20	99.4	46.1
Purkinje cells	2	1	97.8	42.5
Pyramidal Neurons	3	46	81.7	47.1
Mature oligodendrocytes	4	21	72.6	10.2
GABAergic interneurons, PV+	5	59	67.2	12.2
GABAergic interneurons, SST+	6	56	45.5	15.3
Tyrosine hydroxylase expressing	7	51	44.6	26.3
GABAergic interneurons, SST+	8	57	43.2	21
Pyramidal neurons, Callosally projecting, P14	9	40	42	56.4
GABAergic interneurons, VIP+	10	54	32.3	10.5
GABAergic interneurons, PV+	11	64	22.8	29.1
Pyramidal neurons	12	47	9.6	26
Pyramidal neurons	13	45	7.9	35
Drd2+ medium spiny neurons	14	16	5.7	10.9
Pyramidal neurons, corticotectal, P14	15	44	4.3	20.8
Pyramidal neurons	16	48	3.5	18.5
Pyramidal neurons	17	49	0.7	15.1
Pyramidal neurons	18	7	0.6	11.8

Only cell types for which the cosine similarity is larger than 10% are shown.

**Figure 2:**
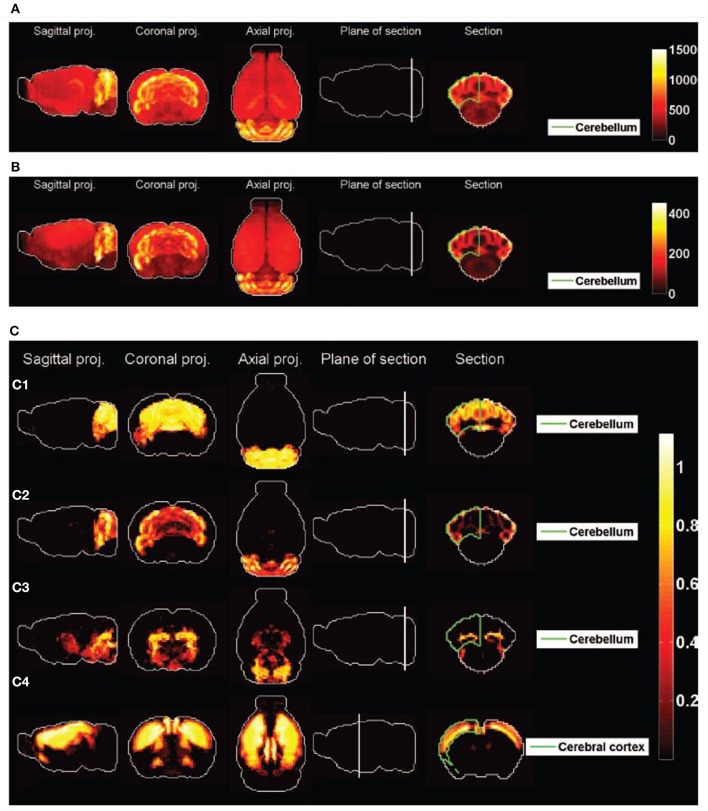
**Heat maps of gene-expression of gene cliques, and of density profiles of cell types**. **(A)** Heat map of the sum of expression energies of the 33 genes in the clique *C*_1_. **(B)** Heat map of the sum of expression energies of the 6 genes in the clique *C*_2_. **(C)** Heat maps of brain-wide densities (denoted by ρ_*t*_ for cell type labeled *t*) of cell types estimated based on the model of Equation (5), for Purkinje cells (C1, labeled *t* = 1), granule cells (C2, labeled *t* = 20), cerebellar mature oligodendrocytes (C3, labeled *t* = 21), and cortical pyramidal neurons extracted from layer 5 (C4, labeled *t* = 46). These four cell types are the ones that are ranked the most highly by statistical significance of similarity to either of the cliques 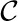
_1_ and 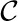
_2_ (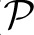
_*R*_ > 80% in Tables 1, 2).

Figure 2 shows heat maps of the expression profiles of the two cliques and of the density profiles of these four cell types. The expression profiles of both cliques highlight the cerebellum, but they are non-zero in many more voxels than any of the densities of cell types illustrated in Figures 2C1–C4. These densities are highly concentrated in the cerebellum (indeed the corresponding cell-type-specific samples were extracted from the cerebellum, see Rossner et al., 2006 for Purkinje cells, see Doyle et al., 2008 for granule cells and mature oligodendrocytes), with the exception of the pyramidal neurons (labeled *t* = 46) which are highly localized in the cerebral cortex (the corresponding cell-type-specific samples were extracted from the layer 5 of the cerebral cortex, see Sugino et al., 2005).

**Figure 3:**
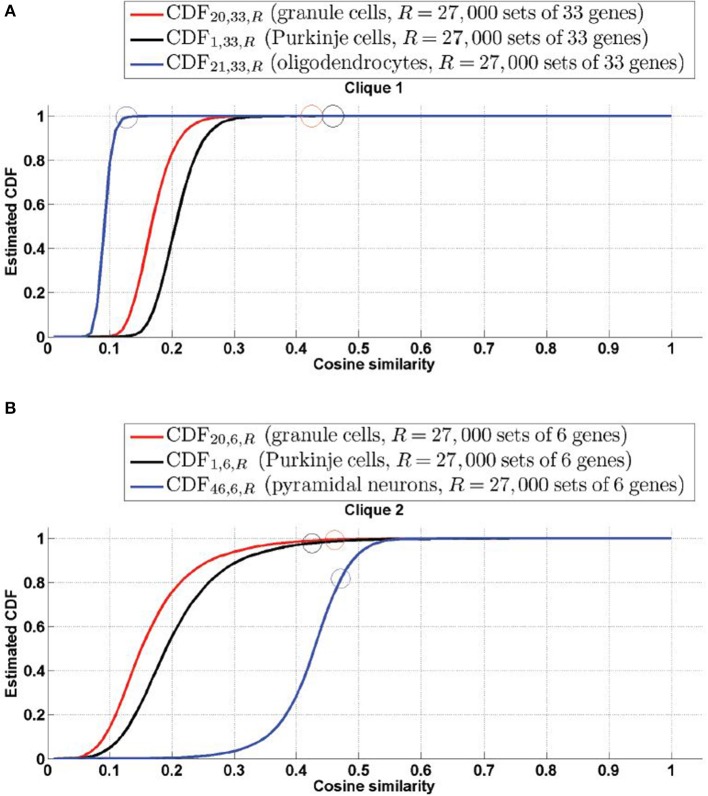
**Simulated cumulative distribution functions (CDFs) of cosine similarities between gene-expression of cliques and the estimated density profile of the three cell types with most significant cosine similarity (granule cells and Purkinje cells for both cliques, along with mature oligodendrocytes for Clique 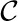
_1_ and pyramidal neurons for Cliques 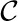
_2_, as per Tables 1, 2)**. The values of the CDFs at the cosine similarities ψ(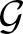, *t*), for clique labeled 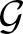 and cell type labeled *t*, are plotted as colored circles. The plots show that granule cells and Purkinje cells both sit extremely comfortably at the top of the distribution of cosine similarities to the expression of both cliques. **(A)** 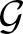 = 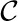
_1_, **(B)** 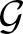 = 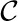
_2_.

The cell-type-specific sample of granule cells (labeled *t* = 20) is the only cell type that has a score higher than 99% in both cliques. Figure 2 shows plots of the simulated CDFs of the cosine similarities between the top three cell types by significance and sets of genes of the same size as 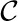
_1_ (Figure 3A) and 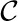
_2_ (Figure 3B). One can observe that both granule cells and Purkinje cells sit more comfortably at the top of the distribution than the cell type ranked third by statistical significance, especially for clique 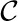
_2_.

**Figure 4:**
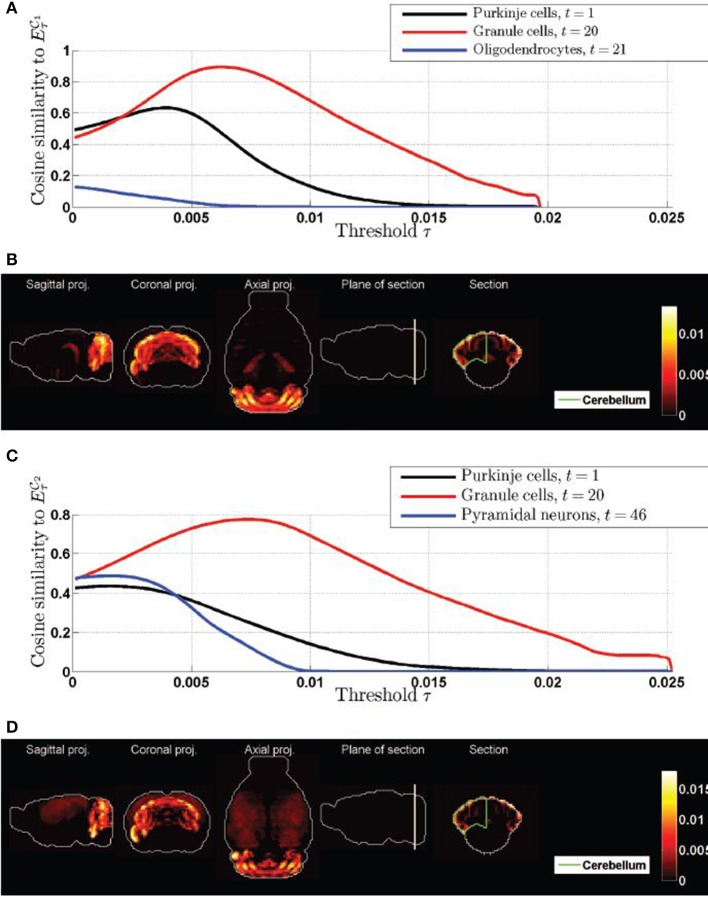
**Cosine similarities of thresolded gene expression energies of cliques, as a function of the threshold. (A)** Plot of ψ_τ_(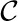
_1_, *t*) as a function of τ for the top three cell types in Table 1. **(B)** Heat map of the expression energy of clique 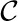
_1_ at the value of the threshold τ for which ψ_τ_(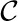
_1_, 20) is maximum. **(C)** Plot of ψ_τ_(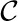
_2_, *t*) as a function of τ for the top three cell types in Table 2. **(D)** Heat map of the expression energy of clique 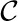
_2_ at the value of the threshold τ for which ψ_τ_(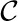
_2_, 20) is maximum. Compare the two coronal sections to the one of the density of granule cells in Figure 2C2.

We therefore need to vary the contrast in the presentation of the expression patterns, in order to decide in which sense, if any, the density profiles of granule cells and Purkinje cells are highlighted differently by the cliques 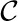
_1_ and 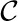
_2_. We computed the cosine similarities between the thresholded expression profiles of each of the two cliques of interest, and the top-three cell types by significance (found in Tables 1, 2), as defined by Equation (12). The values are plotted as a function of the threshold in Figures 4A,C (the expression profiles of the cliques are *L*^2^-normalized so that thresholding parameter τ interpolates between the minimum and maximum value of each of them, and stays in the same range). Granule cells present a peak for both cliques (Purkinje cells do only for the clique 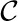
_1_, but at a lower value of the threshold, and the peak is lower, even though Purkinje cells start from a larger similarity to the clique 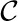
_1_ than granule cells before any threshold is applied). On the other hand, the thresholding procedure lowers the similarity between both cliques and the third cell type returned by the statistical analysis (oligodendrocytes for clique 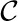
_1_ and pyramidal neurons for clique 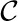
_2_. Moreover, Figures 4B,D) shows heat maps of the expression profiles of both cliques, at the values corresponding to the peak of cosine similarity to granule cells. Indeed the coronal sections through the cerebellum exhibit the characteristic layered, hollow profile of the density of granule cells observed in Figure 2C2, which confirms that the granular layer is highlighted with more intensity by the cliques than the Purkinje layer. Maximal-intensity projections of the thresholded expression profiles exhibit residual expression in the cortex for clique 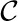
_2_, and to a lesser extent in the hippocampus for clique 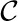
_1_ (but it should be noted that genes are more highly expressed in the hippocampus than in any other region of the brain on average in the coronal ABA).

We therefore conclude that the gene expression profiles of the two cliques of genes in this study highlight the cerebellum with more intensity in the granular layer than in the Purkinje layer, but these two neuroanatomical structures are by far the most exceptionally similar to the expression profiles of the cliques.

### 3.2. Pairs of cell types with exceptional cosine similarities to expression of cliques predominantly involve one cortical and one cerebellar cell type

If we do not threshold the expression profiles of the cliques, they have a non-zero value in the cerebral cortex, albeit lower than in the cerebellum (Figures 2A,B). This combination of cortical and cerebellar expression is not achieved by any of the cell types in our data set, even those that are singled out by our statistical analysis of cosine similarity (as can be checked by visual inspection of Figures 2C1–C4). This compels us to explore better fittings of the expression of the two cliques 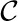
_1_ and 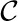
_2_ using more cell types.

**Figure 5:**
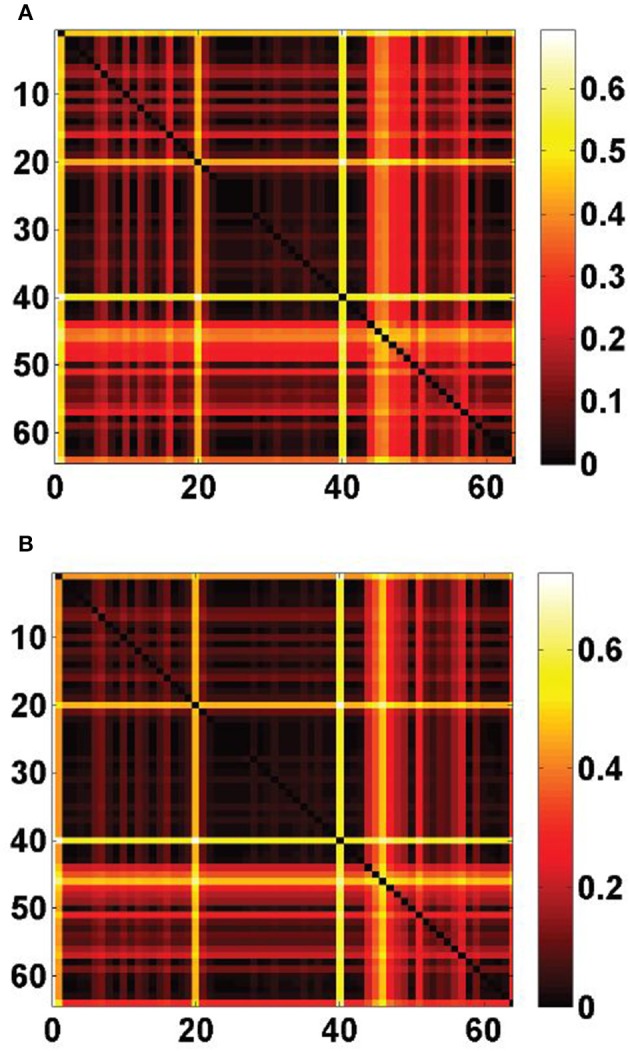
**Optimal cosine similarities between cliques of ASD-related genes and pairs of cell types drawn from the fitting panel of** ***T*** **= 64 cell-type-specific transcriptomes, defined in Equation (17). (A)** Heat map of the matrix ψ(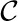
_1_, *t*_1_, *t*_2_), for 1 ≤ *t*_1_, *t*_2_ ≤ 64. **(B)** Heat map of the matrix ψ(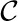
_2_, *t*_1_, *t*_2_), for 1 ≤ *t*_1_, *t*_2_ ≤ 64. Striking horizontal and vertical lines correspond to the labels *t* = 1 (Purkinje cells), *t* = 20 (granule cells), that are returned by the analysis of similarity between cliques and single cell types, but also to the label *t* = 40 (pyramidal neurons, calosally projecting).

We computed the optimal cosine similarity scores defined in Equation (17) for the *T*(*T* − 1)/2 = 2016 possible pairs of cell types from our data set (the results are plotted in matrix form as a heat map on Figure 5). Many of the maxima visibly involve the cell type labeled *t* = 40 which consists of pyramidal neurons, calosally projecting. This cell type also gave rise to high values of cosine similarity between single cell types and both cliques (Figure 1). However, the values 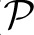
_*R*_(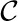
_1_, 40) = 0.4% (rank 59 out of 64) and 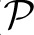
_*R*_(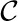
_2_, 40) = 42% (rank 25 out of 64) reflect the fact the values of the cosine similarities to ρ_40_ are biased upwards by the large support of ρ_40_. Other strinking horizontal and vertical lines in the heat maps of Figure 5 correspond to cell types that were already singled out by the above statistical analysis of cosine similarities to single cell types.

For some pairs of cell types, the optimized cosine similarity between a clique of genes and a linear combination of the densities of cell types labeled *t*_1_ and *t*_2_ is not only larger than the similarities with individual density profiles ψ(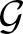, *t*_1_) and ψ(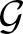, *t*_2_), but it is also larger than the maximum of all the cosine similarities to a single cell type, whose values for the two cliques in this study are:

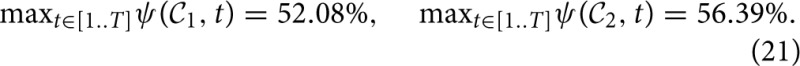



**Figure 6:**
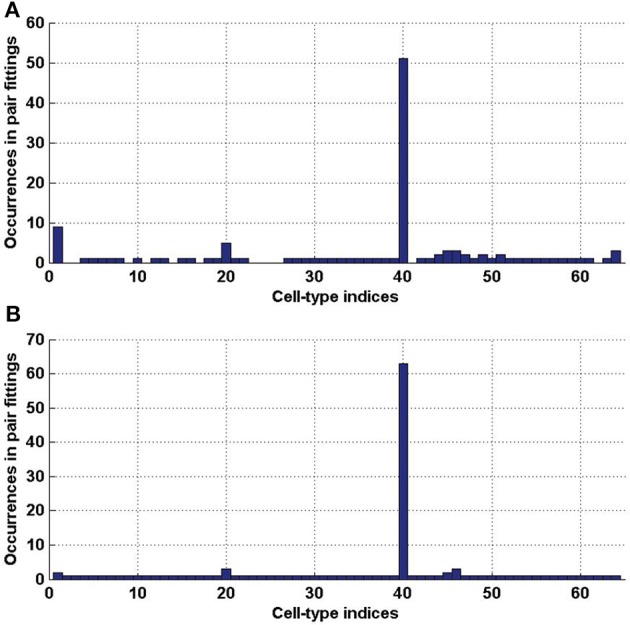
**Histograms of the indices of cell types (in [1..64]), that are involved in a pair of cell types with a better cosine similarity to the expression profile of a clique than any single cell type. (A)** For clique 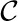
_1_ (total number of elements 124). **(B)** For clique 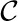
_2_ (total number of elements 132).

**Figure 7:**
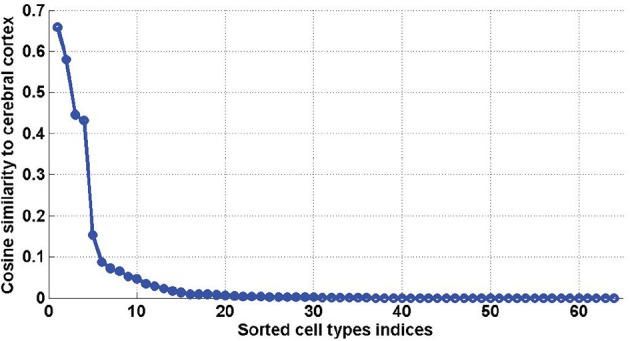
**Sorted values of the cosine similarities between density profiles of cell types and the cerebral cortex** (defined in Equation 20, for ω = cerebral cortex). The first four values, which stand out, correspond to *t* = 40, *t* = 46, *t* = 47, *t* = 45, which are all pyramidal neurons extracted from the cerebral cortex.

The sets of such pairs of cell types (denoted by 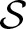
^better^(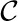
_1_) and 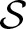
^better^(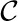
_2_) in Equation 16) consist of 62 and 66 elements, respectively for cliques 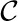
_1_ and 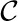
_2_ (which represents 3.08% and 3.27% of the 2016 distinct possible pairs of cell types from our data set). We counted the occurrences of each of the cell types in these special pairs and presented the result in histograms (Figure 6). It appears from both histograms that cell type labeled *t* = 40, plays a special role. This cell type was extracted from the cerebral cortex, and indeed its estimated density profile ρ_40_ is highly localized in the cortex. Moreover, this cell type is the one that has the highest cosine similarity to an ideal density χ_cortex_ that would be uniform in the cerebral cortex and zero elsewhere (see Equation 20 with ω chosen to be the cerebral cortex). A sorted plot of the cosine similarities between estimated density profiles of cell types and χ_cortex_ is presented on Figure 7, showing that four classes of cortical pyramidal neurons stand out, the first of which is labeled *t* = 40.

**Figure 8:**
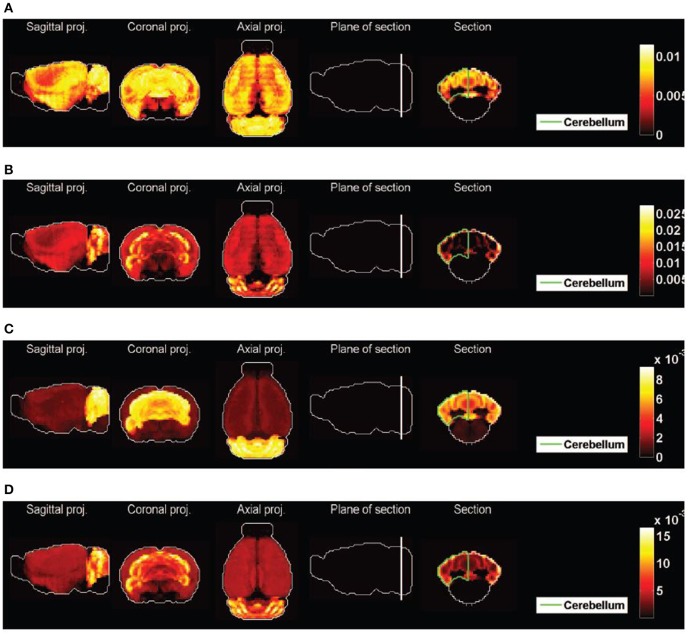
**Heat maps of best-fitted sums of densities of cell types. (A)** Purkinje cells (*t* = 1) and pyramidal neurons (*t* = 40), best fit to clique 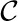
_1_, second best fit to clique 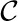
_2_. **(B)** Granule cells (*t* = 20) and pyramidal neurons (*t* = 40), best fit to clique 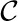
_2_, second best fit to clique 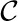
_1_. **(C)** The average of the 13 pairs of cell types illustrated in Table 2A for clique 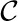
_1_. **(D)** The average of the 5 pairs of cell types illustrated in Table 2B for clique 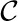
_2_.

Again, for the best fits to pairs of densities of cell types, we have to estimate the probability of obtaining the same results by chance. For each of the cliques, we ran a Monte Carlo simulation of the similarity scores to the 62 and 66 optimal combinations of cell types returned by the above analysis (this simulation is the extension of the quantity *P*_*R*_ of Equation (10) to two cell types, see Equation 18). The combinations of pairs of cell types which have higher cosine similarity to one of the two cliques 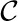
_1_ and 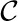
_2_ with probability larger than 99 % are presented in Table 3. They consist of 13 and 5 pairs of cell types, respectively, and the highest-ranking pairs for both cliques (by value of cosine similarity) contain the pyramidal neurons labeled *t* = 40, along with granule cells or Purkinje cells (see Figures 8A,B). For a heat map of the two combinations of cell types presented at the top of Tables 3A,B, see Figures 8A,B, where a distinct cortico-cerebellar pattern appears.

**Table 3:** **Tables of pairs of cell types with higher cosine similarity to a clique of ASD-related genes than any single cell type, for which the value of 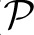
**_***R***_**(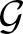,** ***t*****_1_,*****t*****_2_) is larger than 99 percent. (A) For clique 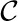
_1_, 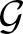 = 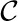
_1_. (B) For clique 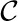
_2_, 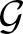 = 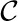
_2_**.

**Index *t*_1_**	**Index *t*_2_**	**Cell type labeled *t*_1_**	**Cell type labeled *t*_2_**	**α^*^_ 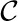 _1__*t*_1_,*t*_2_**	**ψ( 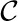 _1_, *t*_1_, *t*_2_), (%)**
40	1	Pyramidal neurons, callosally projecting, P14	Purkinje Cells	(0.521, 0.459)	69.4
40	20	Pyramidal neurons, callosally projecting, P14	Granule Cells	(0.521, 0.424)	67.1
46	1	Pyramidal neurons	Purkinje cells	(0.379, 0.459)	59.5
45	1	Pyramidal neurons	Purkinje cells	(0.366, 0.459)	58.7
64	1	GABAergic interneurons, PV+	Purkinje cells	(0.35, 0.456)	57.7
46	20	Pyramidal neurons	Granule Cells	(0.379, 0.424)	56.9
45	20	Pyramidal neurons	Granule Cells	(0.366, 0.424)	56
64	20	GABAergic interneurons, PV+	Granule cells	(0.351, 0.422)	55.1
20	1	Granule cells	Purkinje cells	(0.295, 0.35)	53.4
51	1	Tyrosine hydroxylase expressing	Purkinje cells	(0.266, 0.458)	53
47	1	Pyramidal neurons	Purkinje cells	(0.264, 0.459)	52.9
44	1	Pyramidal neurons, corticotectal, P14	Purkinje cells	(0.251, 0.458)	52.3
49	1	Pyramidal neurons	Purkinje cells	(0.248, 0.459)	52.1
**Index *t*_1_**	**Index *t*_2_**	**Cell type labeled *t*_1_**	**Cell type labeled *t*_2_**	**α^*^_ 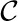 _2__*t*_1_,*t*_2_**	**ψ( 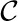 _2_, *t*_1_, *t*_2_), (%)**
40	20	Pyramidal neurons, callosally projecting, P14	Granule cells	(0.564, 0.461)	72.8
40	1	Pyramidal neurons, callosally projecting, P14	Purkinje cells	(0.564, 0.425)	70.6
46	20	Pyramidal neurons	Granule cells	(0.471, 0.461)	65.9
46	1	Pyramidal neurons	Purkinje cells	(0.471, 0.425)	63.4
45	20	Pyramidal neurons	Granule cells	(0.35, 0.461)	57.9

This reflects the fact that the combination of the cerebral cortex and cerebellar cortex (with relative weights specified by the optimal coefficients given in the fifth columns of Table 3) is highly similar to the expression profiles of the two cliques. Moreover, 9 out of the 13 pairs in Table 3A and all the pairs in Table 3B contain a pyramidal neuron (and all pairs contain either Purkinje cells or granule cells). We therefore conclude that allowing one more cell-type-specific degree of freedom to fit the expression profiles of both cliques gives rise to a predominant contribution from pyramidal neurons, all of which are cortical except the hippocampal cell type labeled *t* = 49, and all of which had a much lower statistical significance as single cell types. It can be noted that the four pyramidal neurons with largest similarity to the cerebral cortex (which stand out on Figure 7) are all represented in Table 3.

The averages of all the pairs of cell types returned by our analysis are plotted as heat maps on Figures 8C,D, which can be visually compared to the heat maps of Figures 2A,B. The effect is much better than for any of the heat maps Figures 2C1–C4, even though the heterogeneity of the expression of clique 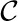
_2_ across the cerebral cortex is not reproduced.

## 4. Discussion

Our computational analysis shows that among the cell types collated in Okaty et al. (2011) and analyzed together with the ABA in Grange et al. (2014), the similarity of the expression of both cliques 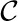
_1_ and 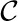
_2_ to granule cells and Purkinje cells is larger than the similarity of more than 97% of the cliques of the same size. These two cell types are the only cell types in our data set to have this property. The statistical significance of the similarity to the spatial density of granule cells is larger than the one of Purkinje cells for the clique 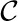
_2_, but Purkinje cells still stand out together with granule cells (which makes sense with the involvement of Purkinje cells in autism discovered in post-mortem studies Skefos et al., 2014). This completes the observation made in Menashe et al. (2013) based on visual inspection of the Purkinje and granular layers of the cerebellar cortex. Granule cells (and not Purkinje cells) may be present in some superficial voxels in which both cliques are highly expressed (see the coronal sections in Figure 2), but as brain-wide neuroanatomical patterns, granule cells and Purkinje cells are both exceptionally similar to the expression profiles of the two cliques in this study. The spatial resolution of the voxelized ISH data of the mouse ABA (200 microns) complicates the separation between granule cells and Purkinje cells, which we attempted here by our thresholding procedure, due to the extreme difference in size between the two cell types. Granule cells and Purkinje cells may be present in the same voxel, and registration errors are therefore much larger in scale of a granule cell than in scale of a Purkinje cell. An interesting direction for a deeper analysis can be found in Ko et al. (2013) and Li et al. (2014), where image series rather than voxelized data are used.

The values of the cosine similarities are not necessarily ranked in the same order as the statistical significances (indeed their values are not decreasing in the fourth columns of Tables 1, 2, which are organized by decreasing order of significance). This is related to the fact that the cosine similarity is biased in favor of cell types present in a larger number of voxels (for example pyramidal neurons, labeled *t* = 46, have a larger support, at 8980 voxels, than granule cells, at 3351 voxels). So, if a clique of genes has a large support (which is the case of both cliques in this study, which have non-zero expression in more than 98% of voxels), it can have a larger cosine similarity to pyramidal neurons than to granule cells, but its similarity to granule cells may be more statistically significant. This is the case for clique 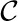
_2_, and the fact is illustrated in more detail on Figure 3B, where it is clear that the similarity between pyramidal neurons (labeled *t* = 46) and clique 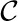
_2_, albeit larger than the value for granule cells and Purkinje cells, sits lower in the distribution of cosine similarities. Our probabilistic approach is therefore a necessary complement to the computation of similarities.

However, two more transcriptomes of Purkinje cells are present in our data set (labeled *t* = 25 and *t* = 52), and they do not stand out in our analysis (their cosine similarity to cliques 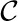
_1_ and 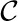
_2_ are 0 for *t* = 25 and 9.2 and 9.5% for *t* = 52, respectively), even though these three transcriptome profiles are close to each other. The difference in cosine similarities is due to the fact that the density profiles ρ_25_ and ρ_52_ are much sparser than ρ_1_, especially in the cerebellum. When fitting the cell-type-based model (Equation 5), similar profiles compete against each other, and the sample *t* = 1 wins in most cerebellar voxels. In Grange et al. (2014), we checked that keeping only one sample of Purkinje cells (*t* = 52, chosen for further numerical exploration as it was independently estimated in Okaty et al. (2009) to be less contaminated by other cell types) and refitting the model yields to similar results as the complete data set, except for the density ρ_52_, which inherits most of the density from ρ_1_. We reran the analysis that returned Tables 1, 2 using these refitted densities, and found that the remaining Purkinje cells occupies the rank of *t* = 1 (with scores 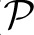
_*R*_(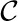
_1_, 52) = 98.8% and 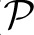
_*R*_(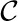
_2_, 52) = 96.5%, respectively), while the other ranks are conserved. Restricting the number of cell types in the panel therefore yields results compatible with the hierarchical nature of cell types. On the other hand, it is crucial to keep a number of genes that is large enough to sample a large subspace of the span of the columns of the matrix *E* in voxel space In Grange et al. (2014), we simulated a thalamic cell type by choosing the 200 genes that are are the most expressed in the thalamus, and constructing a fictitious transcriptome in which the expression of these genes is higher than average. This was shown to be enough to transfer the thalamic density from *t* = 52 to this cell simulated cell type. Hence the signal in a small fraction of a data set can control the competition between two cell types. However, the presence of all the other genes in the data set is necessary to ensure that the densities of other cell types are stable under the inclusion of the simulated cell type, and the (possibly small) sets of genes that control the competition between cells vary from cell to cell.

The robustness of the neuroanatomical density patterns of cell types was shown in Grange et al. (2014) to vary between cell types, but the most unstable spatial density profiles tend to be the sparsest (the *T* cell types were ranked by deceasing stability against subsampling of genes). The cell types that stand out in our results are not among the sparsest ones, as they exhibit striking neuroanatomical patterns. To investigate the stability of our results against the exclusion of cell types, we refitted the model of Equation (5) to a panel of cell types including only the 23 cell types ranked highest for stability (this rank was chosen as it is the lowest rank among those of the 4 distinct cell types presented in Figure 3. The estimated CDFs are stable after refitting (and the values corresponding to statistical significance are within one percent of the values estimated from the full panel).

Moreover, some of the densities of cell types estimated computationally in Grange et al. (2014) can be combined pairwise in order to match the expression of ASD-related cliques of genes better than any single cell types. The optimal combinations we worked out reconcile the involvement of the cerebellum in ASD and the role of the cerebral cortex which had been thought to be predominant. In fact, the use of pairs of cell types to fit the expression of cliques singles out pairs of cell types consisting of one pyramidal neuron and either granule or Purkinje cells. It would be interesting if this association between cortical and cerebellar neurons could be related to connections between the cerebellum and the cerebral cortex (Oh et al., 2014). The improvement of the similarity scores brought by considering pairs of cell types, within this still relatively modest cell-type-specific data set consisting of less than 100 cell types, while a complete taxonomy of neuronal cell types could well be more detailed by orders of magnitude, indicates that the cell-type-specificity of ASD needs multiple genes and multiple cell types (beyond pairs) to be worked out.

One may wonder if our method is not circuitous, compared to the one of Menashe et al. (2013) (in which cosine similarities are computed to estimate the similarity between the spatial expression profile of a clique and a region of the brain defined by classical neuroanatomy and not by gene-expression data). In the present study, given that the expression of a clique of genes is included in the data set that has been used to fit the model of Equation (5). Taking the entire set of genes in the coronal ABA into account allows one to stabilize the results in the sense that we do not need to select genes that are over-expressed in one cell type relative to the others (which choice would have to be refined whenever the set of cell-type-specific transcriptome is modified), and the optimization procedure is equivalent to a competition between cell-type-specific transcriptome profiles. As a numerical experiment, we refitted the model of Equation (5) twice for each clique, using only the genes in the clique the first time, and using its complement the second time. Given that the two cliques of genes of genes contain only 1.1 and 0.2% of the coronal atlas, with expression profiles exceptionally between each other and to the cerebellar cortex, the results of the first refitting cannot detect densities of non-cerebellar cell types, while the results of the second refitting is very close to the original results. As the cliques are small enough not to contain all the genes that are over-expressed in cerebellar cell types, the study of cosine similarities is not too circuitous.

Our analysis shows that the gene-based approach of the ABA and the cell-based approach of the transcriptional classification of cell types in the brain can be combined in order to quantify the similarity between expression patterns of condition-related genes and the spatial density of cell types, even though the region-specificity of transcriptomes of cell types is only accessible computationally. Our results are limited by the paucity of the cell-type-specific data, since the number of transcriptionally distinct neuronal cell types is presumably much larger than 64. However, the classification of cell types is a hierarchical problem, and it is plausible that granule cells and Purkinje cells branch early from each other (and from cortical pyramidal neurons and oligodendrocytes) in the classification, which makes the available data set reasonably effective as a first draft in the context of this study. The computational methods we devised can be easily reapplied when more cell-type-specific microarray data become available. Moreover, alternative measures of similarity can easily be substituted to the cosine similarity, without modifying the analysis of statistical significance and contrast, or the number of random draws dictated by Hoeffding's inequality.

Within data sets of the mouse model organism, the Allen Atlas of the developing mouse brain (http://developingmouse.brain-map.org/) could be used to detect stage-specific changes in expression profiles, as the development of ASD is known to take place in early developmental stages of the brain. However, the current data sets do not allow to repeat the fitting of the model, as the developmental atlas is not co-registered and voxelized, moreover most of the cell type-specific transcriptomes come from adult mouse brains. One can note from Grange et al. (2014) that corticospinal neurons from non-adult mice fit poorly, which could be traced to late maturation of these neurons.

The translation of results from the mouse model to humans is extremely challenging, even though the ABA of the human brain has been released (Hawrylycz et al., 2012), because the human atlas cannot be voxelized, due to the size and paucity of the specimens.

### Conflict of interest statement

The authors declare that the research was conducted in the absence of any commercial or financial relationships that could be construed as a potential conflict of interest.

## Footnotes

^1^which at the time were available as preprint: Grange et al. (2013). Cell-type-specific microarray data and the Allen atlas: quantitative analysis of brain-wide patterns of correlation and density. arXiv preprint arXiv:1303.0013. The correspondence between cell types and their integer labels is the same as in Tables 64, 65 of this preprint. See also the extended discussion of Grange et al. (2014). Cell-type-specific transcriptomes and the Allen Atlas (II): discussion of the linear model of brain-wide densities of cell types. arXiv preprint arXiv:1402.2820.

^2^These data were downloaded from the ABA website http://mouse.brain-map.org, and are included in the MATLAB toolbox with which the results of the present study were obtained. See the current version of the manual and download link in Grange et al. (2012).

^3^or any other measure of similarity.
